# Recent evidence on visual-spatial ability in surgical education: A scoping review

**DOI:** 10.36834/cmej.69051

**Published:** 2020-12-07

**Authors:** Portia Kalun, Krista Dunn, Natalie Wagner, Thejodhar Pulakunta, Ranil Sonnadara

**Affiliations:** 1 Department of Surgery, McMaster University, Ontario, Canada; 2 Faculty of Medicine, Dalhousie University, Nova Scotia, Canada; 3 Office of Professional Development & Educational Scholarship, Faculty of Health Sciences, Queen’s University, Ontario, Canada; 4 Department of Surgery, University of Toronto, Ontario, Canada

## Abstract

**Background:**

Understanding the relationships between structures is critical for surgical trainees. However, the heterogeneity of the literature on visual-spatial ability (VSA) in surgery makes it challenging for educators to make informed decisions on incorporating VSA into their programs. We conducted a scoping review of the literature on VSA in surgery to provide a map of the literature and identify where gaps still exist for future research.

**Methods:**

We searched databases until December 2019 using keywords related to VSA and surgery. The resulting articles were independently screened by two researchers for inclusion in our review.

**Results:**

We included 117 articles in the final review. Fifty-nine articles reported significant correlations between VSA tests and surgical performance, and this association is supported by neuroimaging studies. However, it remains unclear whether VSA should be incorporated into trainee selection and whether there is a benefit of three-dimensional (3D) over two-dimensional (2D) training.

**Conclusions:**

It appears that VSA correlates with surgical performance in the simulated environment, particularly for novice learners. Based on our findings, we make suggestions for how surgical educators may use VSA to support novice learners. Further research should determine whether VSA remains correlated to surgical performance when trainees move into the operative environment.

## Abstract (fr)

**Contexte:**

Il est fondamental pour les chirurgiens en formation de comprendre les liens qui unissent les diverses structures corporelles. Étant donné l’hétérogénéité de la littérature portant sur les habiletés visuo-spatiales (HVS) nécessaires en chirurgie, les éducateurs ont de la difficulté à prendre des décisions éclairées quant à l’enseignement des HVS dans leurs programmes. On a effectué une étude exploratoire de la littérature sur les HVS en chirurgie afin de répertorier la littérature et de cerner des lacunes pouvant faire l’objet de recherches ultérieures.

**Méthodologie:**

On a interrogé des bases de données jusqu’à décembre 2019 à l’aide de mots-clés reliés aux HVS et à la chirurgie. Les articles trouvés ont été évalués de façon indépendante par deux chercheurs pour déterminer leur inclusion à la revue.

**Résultats:**

Au total, 117 articles ont été inclus dans la revue finale. Cinquante-neuf faisaient état d’importantes corrélations entre les tests d’évaluation des HVS et la performance chirurgicale. Cette association est étayée par les résultats d’études en neuro-imagerie. Il n’est pas clair cependant si les HVS devraient faire partie des critères de sélection des résidents et si une formation sur les techniques de visualisation en trois dimensions (3D) est préférable à une formation sur les techniques de visualisation en deux dimensions (2D).

**Conclusions:**

Il semble exister un lien entre les HVS et la performance chirurgicale en contexte de simulation, particulièrement chez les apprenants novices. À la lumière de nos résultats, nous présentons des recommandations sur la façon dont les formateurs en chirurgie pourraient se servir des HVS pour aider les apprenants novices. D’autres travaux de recherche devraient permettre de savoir si les HVS restent reliés à la performance chirurgicale lorsque les stagiaires passent à un environnement opératoire réel.

## Introduction

Visual-spatial ability (VSA) is the capacity to mentally visualize and manipulate objects in 3D space.^1,2^ VSA is important for advancement in science and engineering,^3^ and is assessed for entry into aviation^4^ and undergraduate dental programs.^5^ While these fields are similar to surgery in that they involve a high degree of technical skill,^2^ surgical training programs have not mandated VSA testing prior to entry.^6^ With rapid technological advancements in surgery resulting in new procedures, many trainees are now required to attain competence in both laparoscopic and robotic techniques.^7^ As these techniques involve manipulating surgical instruments with a reduced visual field—challenging depth perception,^8^ hand-eye coordination,^9,10^ and awareness of spatial anatomy^11,12^—there is interest in the relevance of VSA in surgery, and how VSA may be integrated into surgical education.^13–15^

VSA is comprised of many components, each of which can be assessed using a variety of measures (Appendix A). For example, visualization is often assessed through the Mental Rotation Test (MRT), which requires subjects to mentally rotate objects around the vertical and/or horizontal axis.^16^ Spatial orientation is often assessed through the Card Rotation (CR) Test, in which subjects identify whether a card has been rotated or turned over.^16^ There is some evidence that these skills, specifically the ability to mentally rotate objects^17^ and understand the spatial relationships between different structures, are critical for surgical performance.^9,12,17^

Previous reviews present conflicting evidence on whether VSA can be used to predict surgical abilities and/or should be considered in trainee selection. Louridas et al. concluded that visual-spatial tests are promising for performance on a specific subset of surgical tasks; however, more robust research is needed before incorporating visual-spatial test performance into the trainee selection process.^6^ Meanwhile, Maan et al. recommended assessing the VSA of candidates for surgical training.^18^ These reviews provide contrasting suggestions for whether surgical educators should use knowledge of trainee VSA in selection. Further, it is still unclear how educators can use VSA to support trainees within training.

While many papers have explored the relationship between VSA and surgical performance, there are a number of studies that examine VSA outside of the context of trainee aptitude/selection, and there is a lack of synthesized information beyond the two previously mentioned reviews.^6,18^ As such, the aim of this scoping review was to summarize the literature on VSA in surgery and to identify where gaps still exist. We hope this map of the literature will guide future research in VSA and surgery, and assist surgical educators with using VSA to support trainees.

## Methods

To ensure the literature on VSA in surgery was captured effectively, we conducted a scoping review following the framework outlined by the Joanna Briggs Institute (JBI).^19^ The JBI guideline merges the frameworks of Arksey & O’Malley^20^ and Levac et al.^21^^.^

### Stage 1: Identifying the research question

Following a preliminary search, we identified the following question: what is the current state of literature on VSA in surgical education? This question aimed to encompass the breadth of literature on VSA in surgery, allowing us to create a thorough and complete overview of the research in this area.

### Stage 2: Identifying relevant studies

Two independent reviewers (KD and PK) searched the following databases up until December 31, 2019: Ovid-MEDLINE (Ovid-MEDLINE Epub Ahead of Print, In-Process & Other Non-Indexed Citations, Ovid-MEDLINE (R) 1946-December 31 2019, Ovid MEDLINE (R) Daily), Embase (1974-December 31 2019), ERIC-Proquest (1974-December 31 2019), PsycINFO-Proquest (1806-December 31 2019), and Cochrane Library (1999-December 31 2019). Both reviewers independently performed the search and met to discuss any discrepancies. Search terms included variations of the following: *visual-spatial reasoning, skills*, and *abilities*; *surgical performance, education, residency*, and *training*; and various components of VSA (e.g., depth perception and visualization). The search terms were searched as keywords (Ovid-MEDLINE and Embase), ‘anywhere’ (ERIC and PsycINFO), and ‘Title Abstract Keyword’ (default in Cochrane). Boolean terms were used to combine search terms. The full search strategy is shown in Appendix B.

### Stage 3: Study selection

**Figure 1:**
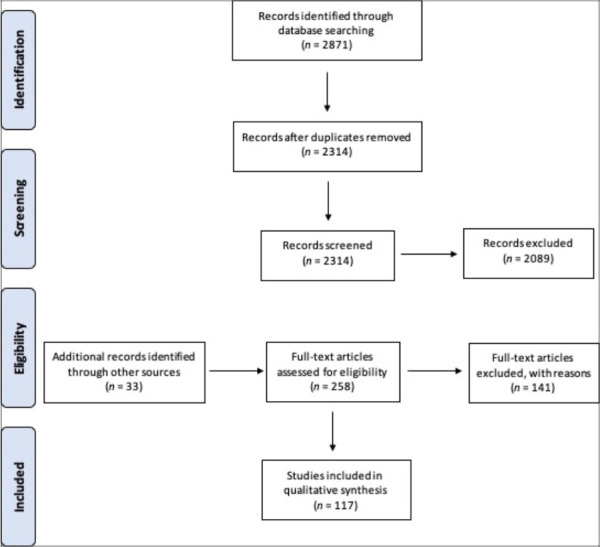
PRISMA flowchart of data following transparent reporting of systematic reviews.^(111)^

The search identified a total of 2871 articles. After 557 duplicates were removed, 2314 articles remained. Following title and abstract screening, 2089 articles were excluded. We hand-searched reference lists of the remaining articles for any relevant articles, identifying 33 additional articles for full-text review. Of the 258 articles reviewed, 117 articles were included for the qualitative synthesis (Figure 1).

Two researchers completed each step of the review process independently, meeting with a third reviewer to resolve any discrepancies. During title and abstract screening, our inclusion criteria followed the participant, concept, and context (PCC) model as outlined by the JBI guidelines.^19^ We did not include studies focused on patients and/or patient outcomes, nor did we include studies written in languages other than English. We included studies whose participants included medical students and surgical residents, but not exclusively so (i.e., studies could also include staff surgeons). All studies focused on the concept of VSA in the context of surgery. As per the JBI guidelines, we included various sources of information for our scoping review, including primary research studies (and/or corresponding abstracts), book chapters, systematic reviews, and narrative reviews. A variety of sources were included to ensure that our review captured the scope of the literature available. If abstracts provided sufficient information, or a corresponding full-text article could be identified, the abstract and/or article was included in the review.

### Stage 4: Charting the data

Two independent reviewers extracted and charted data from the articles included for final synthesis. Data extraction consisted of: author(s), title, year of publication, source of publication, publication type, study design, objective/hypothesis, topic/focus of article, methodology/intervention, outcome(s), key finding(s)/results, conclusion, limitations, and generalizability. As opposed to systematic reviews, which seek only the best available evidence, scoping reviews aim to provide a map of evidence from the literature, and do not require assessment of the quality of evidence gathered.^19^ Thus, we did not assess the quality of the included articles when charting the data.

### Stage 5: Collating, summarising, and reporting the results

The study team synthesized the extracted data and identified major themes using a thematic analysis, which focuses on identifying patterns of meaning from the collected data.^19^ Synthesis was an iterative process, with the study team meeting regularly to discuss findings. Following data extraction, the primary data reviewers (PK and KD) independently grouped articles together based on what each article was exploring. The reviewers (PK and KD) then met with the rest of the research team to discuss their findings, specifically the commonalities and differences among the included studies, until the team reached consensus.

## Results

Of the 117 articles included, there were 90 original research articles, eight narrative reviews, four abstracts, four letters to the editor, four systematic reviews, three methods papers, two book chapters, one meta-analysis, and one editorial. Of the 90 original research articles and four abstracts, 86 described observational studies and eight described experimental studies. All papers discussed the role of VSA for performance in surgery. Based on our thematic analysis, findings from the articles were grouped into four major themes: VSA and surgical performance, neuroimaging studies measuring brain activity during VSA tasks and surgical tasks, VSA aptitude testing, and 2D (monoscopic) versus 3D (stereoscopic) training in surgical education.

### Theme One: VSA and surgical performance

Seventy-five out of 117 articles compared performance on VSA tests to performance during surgical simulation tasks, including laparoscopy (included in 49 articles), endoscopy (included in 14 articles), and 13 other simulation tasks; see supplemental material for a summary of key findings from each article (Tables S1-S3). Medical students and residents were the most commonly studied, with 33 and 31 of the 75 simulation studies including these populations, respectively. Staff and consultant surgeons were included in 19 of the 75 studies.

**Table 1:** Summary of articles comparing VSA to performance on a surgical simulator.

VSA component	Evaluated in *x* articles	No. of articles reporting a significant correlation between VSA and performance on surgical simulators
Visualization	38	28/38^1,7,10,11,16,22,28,29,32,33,39,44,46–48,50–52,54,57,70,74,75,107,112–115^
Spatial Orientation	32	26/32^7,10,16,22–25,27–35,40,45,49,55,69,78,116–119^
Spatial Scanning	10	6/10^26,32–34,49,120^
Other VSA components^*^	28	16/28^16,25,32,34,41,45–47,70,112,121–126^

* Depth perception, flexibility of closure, speed of closure, spatial memory, spatial relation, and perceptual speed

Visualization and spatial orientation were the most common VSA components measured (Table 1), and were significantly correlated with performance on a surgical simulator in 28/38 and 26/32 of studies, respectively. Typically, the studies that found a significant correlation had used the MRT, CR Test, Cube Comparison (CC) Test, PicSOr, or the Hidden Figure Test (HFT) as measures of visualization and/or spatial orientation.^6,22–43^ In addition to improved performance, nine studies reported that individuals with higher visualization skills, as assessed by the MRT-A, performed faster^16,44–49^ and more accurately^50,51^ than individuals with low visualization skills on surgical simulation tasks.

Eleven studies reported that individuals with high VSA scores required less training time to reach proficiency on simulation tasks when compared to individuals with low VSA scores, regardless of which VSA component was evaluated.^4,10,16,32,35,36,46,52–55^ In contrast, seven studies reported no such difference between the training time required to reach proficiency between those with high VSA scores and those with low VSA scores.^31,50,56,57,57–60^ Further, five studies did not find an association between VSA scores and simulation performance,^61–65^ while one found an association between only perceptual speed, and not visualization or spatial orientation, and simulation performance.^41^ One study also explored the influence of gender on learning laparoscopic knot tying, reporting that males had increased visualization following an educational intervention (cognitive imaging) compared with females.^66^ When comparing VSA scores of novices with experienced surgeons, four studies suggested there were no significant differences between the two groups,^33,37,67,68^ whereas four articles reported novices outperforming experts on VSA tasks.^58,69–71^ Lastly, Hegarty and colleagues^72^ and Abe and colleagues^39^ reported high-spatial individuals are at an advantage early in training; however, the effects of spatial abilities may diminish with training. Yet, multiple papers reported a significant correlation between visualization and surgical simulation performance for both novice trainees^11,58,70,73^ and those further into residency training.^22,47,48,54,55,57,74,75^

Two out of the 117 articles compared performance on VSA tests to clinical performance.^76,77^ Hoan and colleagues^76^ found a significant positive correlation between performance on the CR Test and gynecology surgical skills. However, the CR Test scores also increased significantly from year one to year two of the study, suggesting that the CR Test may not be a reliable measure of VSA as it is subject to change with experience.^76^ Selber and colleagues^77^ used the Structured Assessment of Microsurgery Skills (SAMS), which includes ratings of VSA. There was a significant improvement in skills of plastic surgery trainees over the four-month period of the study, including an improvement in the SAMS measure of VSA.^77^

Lastly, five studies explored the relationship between VSA and surgical performance (either in simulated or clinical environments) and measured VSA as part of an assessment of surgical performance rather than measuring a specific VSA component (e.g., visualization via the MRT). Three studies used five-point rating scales to measure visuo-spatial ability,^77^ spatial orientation,^78^ and depth perception^79^ as specific domains on assessments of trainee competence. Due to the nature of the assessments, better VSA was correlated with increased overall performance on the simulators.^78,79^ Two other studies measured VSA via motion analysis built into endoscopic trainers.^9,80^ As with the VSA domains being built into assessments of performance, motion analysis measures of spatial awareness were directly related to overall performance on the simulators.^9,80^

### Theme Two: Neuroimaging studies correlating VSA and surgical performance

Five experimental studies and one letter to the editor focused on neuroimaging [functional magnetic resonance imaging (fMRI) and functional near-infrared spectroscopy (fNIRS)] during surgical and VSA tasks. Three experimental studies used fMRI, a non-invasive technique that monitors cortical brain activation through hemodynamic responses, to identify which areas of the brain are active during VSA tasks.^14,81,82^ Bahrami and colleagues reported a significant increase in parietal activation^14^ and differences in primary motor cortex activation^82^ during increasingly difficult Fundamentals of Laparoscopic Surgery (FLS) tasks, and Wanzel et al.^81^ reported statistically significant activation of bilateral inferior parietal regions and lateral prefrontal and left middle temporal areas, during an MRT-A task.

Two studies^83,84^ measured cortical activation during minimally invasive surgical tasks using fNIRS, a newer technology that can more accurately determine oxygen consumption^83^ by measuring neuron-related hemodynamics. This technology can be utilized in the operating room (OR), unlike fMRI.^85^ Through this technique, Leff and colleagues^83^ found increased hemodynamics in the fronto-parietal cortical areas both for VSA tasks and minimally invasive grasping tasks. Paggetti and colleagues^84^ found activation of the posterior parietal cortex in tasks that required hand-eye coordination and depth perception.

### Theme Three: VSA aptitude testing

Twenty-four articles discussed VSA aptitude testing for entry into surgical programs (Table S4). Stolk-Vos and colleagues^86^ explored the feasibility of aptitude testing of medical trainees using the Computerized Pilot Aptitude and Screening System (COMPASS). While testing of surgical candidates was deemed feasible, it is unknown whether the COMPASS can predict surgical performance.^86^ A systematic review by Maan et al.^18^ concluded that intermediate- and high-level VSA, specifically on measures of perception, can predict surgical skill acquisition and ability. As such, Maan et al.^18^ suggested that VSA be used to assess candidates for surgical programs. Six other articles supported this notion due to the correlation between psychomotor ability and performance of surgical trainees found in the literature.^4,15,38,87–89^ Two articles describe a suite of tests that can be used to screen candidates.^90,91^ However, thirteen studies suggested further research is required before VSA testing is incorporated into surgical residency applications as there remains some conflicting evidence surrounding VSA and surgical performance.^2,5,13,59,71,92–99^

Further, some studies suggest that spatial abilities may be important for the selection and training of novices^94,96,100^ and intermediate learners,^100^ but the importance of having high VSA may diminish with extensive operative experience.^70,72,96,101,102^

### Theme Four: 2D (Monoscopic) versus 3D (Stereoscopic) training in surgical education

Ten articles discussed 2D versus 3D training as it applies to VSA and surgery (Table S5). Conventional 2D methods provide the image of the scope on a 2D display, whereas 3D methods provide a unique image to each eye, resulting in a 3D view of the surgical field.^12^ While the depth cues afforded by 3D displays are thought to enhance skill acquisition, surgeons may alter their reliance on specific depth cues when performing skills using 2D displays.^103^ Of the nine studies discussing 2D versus 3D displays, four reported a benefit associated with using 3D displays in surgical simulation.^8,12,51,53^ This was particularly true for learners with low VSA, as it provided additional information to these learners when compared to 2D displays.^8,12,51,53^ However, four of the nine articles reported no significant differences in training on 2D versus 3D displays for performance on a variety of tasks, including perception of surgical images,^67^ surgical flaps,^51^ and the McGill Inanimate System for Training and Evaluation of Laparoscopic Skills (MISTELS).^26,53^ Further, Paggetti and colleagues^84^ found similar differences in posterior parietal activation for hand-eye coordination and depth perception tasks, regardless of whether these tasks were performed in 2D or 3D conditions.

## Discussion

Of the 75 articles measuring the correlation between surgical trainees’ VSA scores and performance on surgical simulators, 59 reported significant correlations. These significant correlations were mainly found in studies that measured visualization and spatial orientation components of VSA. Additionally, the neuroimaging studies using both fMRI and fNIRS reported that the same areas of the brain are active during visualization (as measured by the MRT) and surgical tasks.^14,81,83^ For this reason, we conclude there is evidence to support a relationship between surgical performance and VSA, specifically for measures of visualization and spatial orientation. Since this was a scoping review and quality of studies was not evaluated, we cannot make statements about the strengths of the associations reported in our themes.

There are many ways to measure visualization (e.g., MRT, Paper Folding Test (PFT), Surface Development Test (SDT), Keyhole Test, etcetera) and spatial orientation (e.g., CR Test, CC Test, Stumpf-Fay Cube Comparisons Test, etcetera). However, this review suggests the MRT, CR Test, and/or CC Test have the most evidence when interested in surgical performance. The MRT is a useful measure of surgical simulation performance as individuals must mentally rotate objects,^17^ a task common in surgical procedures. The MRT was also the most frequently studied measure, with authors often reporting significant correlations with performance on surgical tasks. For spatial orientation, the CR and/or CC tests are useful measures, as understanding the spatial relationship between different structures is critical in surgery.^9,12,17^ Further, the CR and CC tests were some of the more frequently studied measures in this review.^22–36,76^

Measuring VSA in surgical trainees is important not only because the results suggest that individuals with higher VSA often demonstrate increased surgical performance,^4,10,16,22,31,32,35,36,44–46,48–50,52,54,55^ but also that those with higher VSA often require fewer training sessions to reach a certain performance point than their peers with lower VSA.^4,10,16,32,35,36,46,52–54^ Future research should aim to determine the scores on VSA tests that may result in educationally relevant differences in learning technical skills. The evidence from this review suggests that trainees with higher VSA compared to their peers may learn surgical skills faster, thus privileging later clinical learning and allowing them to advance beyond their peers. By assessing trainee VSA early on, trainees with lower VSA compared to their peers may be identified, and can be provided with additional support before their technical abilities fall behind that of their peers. In terms of how to support these learners, this review identified conflicting evidence on whether 2D or 3D simulators are the best approach. Three studies reported that training on 3D simulators provides additional information to learners and would especially benefit those with lower VSA.^12,51,53^ However, four studies suggested training on 2D simulators provides similar or superior results to 3D training.^26,51,53,104^ The studies included in this review did not investigate the potential interaction between fidelity of the model and viewing modality (i.e., 2D versus 3D); however, Mistry and colleagues^26^ suggest that additional information provided by a 3D view may be too cognitively demanding for novice learners, especially in a high-fidelity simulation. Future studies should investigate not only whether 2D versus 3D simulators are more effective at supporting learners, but also the potential interaction between simulator fidelity, viewing modality, and level of experience to better understand how 2D versus 3D modalities influence learning. Until then, we suggest training on 2D models is sufficient for surgical education due to the conflicting results on which training modality is superior, and because 2D training resources are less expensive,^105^ more commonly used in the OR, and familiar to trainees.^12^

In sum, our findings suggest there is evidence to support using VSA, specifically measures of visualization (e.g., MRT) and/or spatial orientation (e.g., CR or CC tests), as an adjunct to 2D simulator training. However, whether VSA should be used for trainee selection into surgical residency programs remains undecided. Despite the evidence of a correlation between VSA and surgical performance, there continues to be hesitation to use VSA assessments for trainee selection.^59,92,93,95,106^ This is likely due to conflicting evidence on whether VSA scores are correlated with performance solely during the initial skill acquisition phase, or across all stages of training.^33,37,107^ If VSA is correlated with surgical performance at all levels, it suggests that VSA is a stable characteristic and therefore may be a strong predictor of surgical aptitude. However, if VSA is only correlated with surgical performance in novices, it suggests that VSA may be a fluid, and trainable characteristic, and has the potential to be acquired throughout residency training with practice^3^ We believe that if VSA is a trainable characteristic, high VSA should not be a requirement for those applying to surgical residencies.^12,15,53,108^ To address this concern, future research should focus on correlating VSA to surgical performance across all levels of training. One way to achieve this would be to measure trainees’ performance on a VSA test (e.g., MRT, CC Test, or CR Test) and performance on surgical simulations annually to identify a pattern across years of training. This could further identify the potential to use VSA tests to assist trainees struggling with surgical simulation performance.

Hesitation to use VSA assessments for trainee selection may also be due to studies comparing VSA with performance in simulation, rather than performance in the clinical environment. While simulation is certainly recognized as an important adjunct to surgical education—giving trainees the opportunity to develop skills before interacting with patients^109^ and the ability to practice in a lower-risk environment^109^—there remains conflicting evidence on whether skills learned in simulation are transferrable to OR performance.^110^ Our review identified only two studies that explored VSA and clinical performance, neither of which found compelling evidence for a role of VSA in surgical performance.^76,77^ Further, evidence suggests that VSA is not correlated with surgical performance of experienced surgeons, who consistently work in the clinical environment.^58,69,70^ Future research should investigate the correlation between VSA, specifically visualization and/or spatial orientation, and trainee performance in the OR. By using technology such as fNIRS, areas of the brain that are activated during surgical tasks in the OR could be identified and correlated to the activation seen while trainees completed visualization and/or spatial orientation tests. However, if feasibility (e.g., cost, lack of neuroimaging experts to analyze the data) prevents that from occurring, we suggest future research focuses on identifying which of the skills that correlate to VSA reliably transfer from simulation practice to improved performance in the OR. For example, researchers may begin with identifying whether skills on specific laparoscopic (e.g., MIST-VR, LapSim) or endoscopic (e.g., GI Mentor II) simulators transfer to improved clinical performance, as many studies in our review found significant correlations between VSA and performance on these simulators. This would subsequently allow researchers to focus on correlating VSA components to the specific skills that we have identified, and that are transferable to the clinical environment. Until additional research is conducted on these areas, we do not recommend VSA be used as a selection criterion for surgical programs.

## Limitations

Though this study provides a map to existing literature on VSA in surgical training, it was not a systematic review or a meta-analysis. Statistics could not be reported in tables due to the varied and inconsistent methodologies and analyses across the articles. Also, since full-text articles published outside of the English language were not included, we may have overlooked relevant data published in non-English articles.

## Conclusion

This scoping review investigated the recent literature surrounding VSA in surgery. We identified four themes: VSA and surgical performance, neuroimaging studies correlating VSA and surgical performance, VSA aptitude testing, and 2D (monoscopic) versus 3D (stereoscopic) training in surgical education. From this review, we suggest visualization, specifically the MRT, and spatial orientation, as measured by the CR and CC Tests, may be used to predict trainee performance of surgical skills in simulation. Identifying VSA levels in surgical trainees may also provide educators with the opportunity to identify trainees struggling with surgical performance. Our results suggest additional 2D simulator training may be one way to support these learners, although future research is needed to explore how fidelity and level of training influences this. Additionally, future research is needed to evaluate VSA across different levels of training to determine whether it is a stable or trainable characteristic, and compare VSA to operative performance directly, for which we suggest fNIRS to be a safe and reliable method. Such work will inform whether VSA can be used to assess applicants to surgical residency programs and to support learners currently in training.

### Highlights

VSA scores are correlated with surgical simulation performance of novices in many studies, but it is unclear whether VSA scores are correlated with performance across all stages of training.

Visualization and spatial orientation, as measured by MRT and CR and/or CC Tests, respectively predict performance on surgical simulators.

More research is required to determine whether VSA scores predict performance in the clinical environment.

## Appendices

### Appendix A

**Table:**

VSA Component	Test	Test Description	# Studies
Depth Perception	PicSOr	On a computer screen, subjects are presented with a spinning arrowhead with its point touching the surface of a geometric object (cube or sphere). The task is to maneuver the arrowhead using the computer mouse until its shaft is perpendicular to object surface at the point where it was originally touching. This tests subjects' ability to recover pictorial cues that specify how structures are oriented in virtual pictorial space.	11
Depth Perception	Titmus Stereo Fly Test	Subjects are asked whether a picture of a fly appears to be three-dimensional.	1
Depth Perception	TNO Stereopsis Test	Subjects are asked to identify geometric shapes, some of which are only visible with stereoscopic vision.	1
Depth Perception	Frisby Stereotest	Subjects are asked which of four groups of triangles stand out to them.	2
Depth Perception	Graded Circle Test	Subjects are asked which diamonds appear closer (i.e., stand out).	1
Flexibility of closure/field of closure	Embedded Figures Test (EFT)	A simple geometric figure is shown, then subjects are asked to find the figure embedded or hidden in a relatively complex figure.	6
Flexibility of closure/field of closure	Hidden Figures Test (HFT)	Subject has visual environment filled with complex figures and needs to identify a specific spatial object/figure embedded within such environment.	8
Perceptual Speed	Identical Pictures Test (IPT)	Subjects must compare figures or symbols, or scan an image to find a specific figure or symbol. Time to completion is recorded.	5
Perceptual Speed	Number Comparison Test (NCT)	Subjects must inspect pair of multi-digit numbers and indicate if two numbers are same or different. Time to completion is recorded.	3
Spatial Ability	Wechsler Intelligence Scale for Children–III (WISC-III) Cubes	Subject uses two different colours of cubes to reproduce 12 given figures. The faster the task is completed, the more points given.	2
Spatial Orientation	Card Rotation Test (CR)	Test consists of a 2D drawing of a card cut into an irregular shape. To the right of this irregular shape, there are six 2D drawings of the same irregular card, either rotated or flipped over. Subject must indicate whether the irregular card has been flipped over or rotated.	24
Spatial Orientation	Cube Comparison Test (CC)	Subjects are given cubes with pattern on each side (three sides are visible). Subjects have to identify the cube that matches a target cube.	20
Spatial Orientation	Rotating Shapes Test	Two complex irregular polygonal shapes are presented (one has been rotated). Subjects are asked to identify whether the two shapes are identical, or mirror images of one another, as quickly as possible.	2
Spatial Orientation	Orientation Test	Subjects are presented with 2D images of geometric shapes and are asked to mentally rotate the shapes in 3D space to understand their spatial orientation. Subjects must select correct rotation.	4
Spatial Orientation	Mental Rotation Reaction Time Test	Two complex irregular polygonal shapes are presented (one has been rotated). Subjects are asked to identify whether the two shapes are identical, or mirror images of one another, as quickly as possible.	1
Spatial Orientation	Stumpf-Fay Cube Perspectives Test	In this test, different views of complex tubular figures have to be judged on their rotation with respect to a specific point of view.	1
Speed of Closure	Form Completion Test (FCT)	Subjects must identify objects from incomplete silhouette drawings.	3
Speed of Closure	Gestalt Completion or Closure Test	Drawings of an incomplete picture in black and white representing a part of an object is shown to subjects. Subjects must name the object and speed of which they name the object is measured.	4
Speed of Closure	Snowy Picture Test	Subjects must identify objects that are partly obstructed.	2
Spatial Scanning	Map Planning Test (MPT)	Subjects must plan routes on a map while avoiding barriers, as fast as possible.	11
Spatial Scanning	Maze Test	Subjects must go through a paper maze as quickly as possible while avoiding barriers.	3
Spatial Scanning	Localization Test	Within 3 seconds, subjects must view the “x” located in each of 24 rectangles projected on a screen, and place an “x” mark in the same relative location on an answer sheet.	1
Spatial Relation	Block Touching Test (BTT)	Subjects are presented with a drawing where some blocks are numbered and some are not. Subjects must count the number of blocks touching each of the numbered blocks.	3
Spatial Relation	Spatial Relations Test (SRT)	Subjects mentally reconstruct a 3D object from a 2D pattern and mentally rotate this object in space. Subjects must complete 50 tasks within 25 minutes.	3
Visualization	Form Board Test (FBT)	Subjects are given five small shapes and must select the shapes that together create a predetermined larger shape.	6
Visualization	Keyhole Test	Subjects are given a 2D shape and are asked to determine which hole the shape will be able to pass through.	1
Visualization	Paper Folding Test (PFT)	Subjects are shown a paper folded two or three times with a hole punch in it. Subjects must then select the drawing that correctly depicts where the hole would be if paper were unfolded.	7
Visualization	Purdue Spatial Visualization Test	Subjects must mentally rotate an image to see which geometric figures it could correspond to.	5
Visualization	Mental rotation task A (MRT-A)	Figures requiring mental rotation around vertical axis.	41
Visualization	Mental rotation task C (MRT-C)	Figures requiring mental rotation around vertical and horizontal axis.	2
Visualization	Surface Development Test (SDT)	Each question shows how a 2D piece of paper might be cut and folded to make a 3D shape. Dotted lines or numbers on the 2D diagram show where paper is folded. Subjects are asked to match to numbers on the 2D diagram, to the letters on the 3D shape.	9
Visualization	3D Blocks Game	Computer-based game where subjects mentally rotate blocks around x, y and z axes until blocks fall into a pit.	1
Visual Problem Solving	Matrix Reasoning Test	Subject are given an impartially drawn shape and they must fill in the missing shape from number of choices.	1
Visual-motor, organization, visual memory and VS Process	Rey-Figure Test (RFT)	Subjects must copy/draw a figure and repeat five minutes later.	3
Visuo-spatial memory	Corsi block-tapping test	Subjects copy sequences of blocks being tapped.	1

**Table:**

VSA Component	Description
Visualization	Manipulate complex mental representations
Spatial Orientation	Ability to perceive spatial patterns or maintain orientation with respect to objects in space
Flexibility of closure/ field of closure	Identify spatial forms that are specified to the learner in advance in a cluttered visual environment
Perceptual speed	Quickly identify a given shape from number of alternatives
Depth Perception	Ability to perceive depth
Spatial scanning	Speed in exploring visually wide or complex spatial field
Spatial relation	Ability to envision depth and structure of 3D objects depicted on 2D plane
Speed of closure	Write an apparently disparate perceptual field into a single concept
Spatial ability	Spatial ability is the capacity to understand and remember the spatial relations among objects
Visual-motor, organization, visual memory and VS process	Visual memory and recall of objects
Visual Problem Solving	Ability to identify and visualize missing shapes to complete an object formation

### Appendix B. December 31, 2019

**Table:**

Number	Search Terms
1	spatial reasoning
2	visuospatial reasoning
3	visual-spatial reasoning
4	spatial skill^*^
5	visuospatial skill^*^
6	visual-spatial skill^*^
7	spatial abilit^*^
8	visuospatial abilit^*^
9	visual-spatial abilit^*^
10	surg^*^ performance
11	surg^*^ education
12	surg^*^ residen^*^
13	surg^*^ train^*^
14	depth perception
15	flexibility of closure
16	field of closure
17	perceptual speed
18	spatial orientation
19	speed of closure
20	spatial scanning
21	spatial relation
22	visualization
23	visual problem solving
24	1 or 2 or 3 or 4 or 5 or 6 or 7 or 8 or 9
25	10 or 11 or 12 or 13
26	14 or 15 or 16 or 17 or 18 or 19 or 20 or 21 or 22 or 23
27	24 or 26
28	25 and 27
